# Common Pediatric Otolaryngology Procedures: Ergonomic Considerations

**DOI:** 10.7759/cureus.34678

**Published:** 2023-02-06

**Authors:** Nicole L Aaronson, James S Reilly

**Affiliations:** 1 Department of Surgery, Section of Otolaryngology, Nemours Children's Health, Wilmington, USA; 2 Department of Otolaryngology, Department of Pediatrics, Thomas Jefferson University Sidney Kimmel School of Medicine, Philadelphia, USA

**Keywords:** surgeon preference, patient positioning, workplace injury, operating room workflow, pediatric otolaryngology

## Abstract

Background

Otolaryngologists in high-volume children’s hospitals developed their operating room workflow practices based on the factors of safety, efficiency, and surgeon preference. Recent data show important benefits and potential reduced risks of proper ergonomic positioning for surgeons to prevent injury. These data suggest that the current operating room workflow practices, in addition to prior training, should be monitored and hopefully improved for surgeons’ health. Surprisingly, recent studies have suggested the benefits of standing versus sitting on cognitive function.

Objective

This study reports the workflow norms for seven operating procedures in pediatric otolaryngology. We seek to identify 1) surgeon preferences, 2) when practices become norms, and 3) whether procedure positions are associated with surgeon discomfort or injury.

Methods

The Otolaryngology Section of the American Academy of Pediatrics was queried employing a 23-question survey. We included demographic information, reasons for preferences, and surgeon-reported pain. We focused on three workflow issues: 1) length of procedures, 2) site selection (operating room bed vs. transport stretcher), and 3) position of the surgeon (sitting vs. standing).

Results

Sixty-nine American Academy of Pediatrics members completed the survey. The length of the procedure had minimal effect, with 90% sitting for short procedures such as bilateral myringotomy with tubes, myringoplasty, tonsillectomy, and adenoidectomy. All sit for direct laryngoscopy and bronchoscopy. Most stand for the removal of nasal foreign bodies, drainage of neck abscess procedures, and thyroglossal duct cyst excision. Residency training (75%) and personal comfort (81%) were the more frequently cited reasons for preference. Fewer than one in five (16%) reported preexisting neck or back pain, but this doubled (35%) throughout their otolaryngology practice. Conditional distributions of pain showed reports of pain were greater for individuals in practice for over 20 years.

Conclusions

Pediatric otolaryngologists develop their operating room preferences early during residency training. High rates of neck and back pain (35%) may develop during a surgeon’s career. We suggest improved understanding of ergonomics in concert with operating room workflow should be considered during otolaryngology residency training since recent data suggest potential benefits of standing on cognitive function.

## Introduction

Safety in the operating room in pediatrics focuses on proper patient identification, safe positioning to avoid injury, aseptic techniques to prevent infections, and improved surgical outcomes [1]. Efficiency is also important when the volume of surgical cases is high, the duration of cases is short, and rapid room turnover reduces medical costs. However, the position of the surgeon in the operating room has received less scrutiny and may affect efficiency and safety [2].

This study reviews surgeon preferences in seven common otolaryngology surgeries in pediatric hospitals. We seek to identify why certain practices become norms and whether operating room workflow is associated with the length of procedures or surgeon preference. Our survey seeks to discover opportunities for improvement in patient care and provider comfort through changes in behaviors. Recently, data suggesting that “standing improves brain activity” and may have a positive impact on cognitive function has been reported [3]. The New York Times editorialized that “Sitting is the new smoking!” However, there may also be a concern for surgeon stability for fine motor tasks in small spaces like otologic surgery.

## Materials and methods

The American Academy of Pediatrics Section on Otolaryngology-Head and Neck Surgery was petitioned for the use of their mailing list to distribute this survey. The survey was distributed twice via email (March 2020 and April 2020). The survey was considered closed after no new survey completions were noted for two consecutive days.

Seven common pediatric otolaryngology procedures were studied. Demographic information included surgeon height, years in practice, and the number of pediatric cases performed monthly. Questions about preexisting neck or back pain or new onset of pain for the surgeon were developed. Preexisting pain was defined as pain present before otolaryngology training and practice. The survey instrument contains 23 separate fields addressing surgeon preferences (sitting vs. standing) and positioning (operating room bed vs. transport stretcher).

This online survey instrument (Appendix 1) was built and managed using REDCap electronic data capture tool [4,5]. REDCap (Research Electronic Data Capture) is a secure, web-based software platform designed to support data capture for research studies, providing 1) an intuitive interface for validated data capture, 2) audit trails for tracking data manipulation and export procedures, 3) automated export procedures for seamless data downloads to common statistical packages, and 4) procedures for data integration and interoperability with external sources. Exemption for completion of this study was obtained from the Nemours Institutional Review Board.

## Results

About 40% of pediatric otolaryngologists (69/178) completed the survey. Respondents were evenly distributed with respect to years in practice, with a plurality between 11 and 20 years (1-5=23%, 6-10=19%, 11-20=17%, 21-30=26%, > 31 years=15%).

Over 90% of surgeons in our study perform in high-volume settings with > 10 procedures per week and average 41 cases per month (< 20=6%, 21-40=15%, 41-60=32%, 61-80=19%, and > 81=29%). All surgeons perform in a tertiary children’s hospital, and three-quarters (73%) also perform cases in an associated outpatient surgery center.

All surgeons sit for direct laryngoscopy and bronchoscopy procedures. Most (87% to 96%) pediatric otolaryngology surgeons also sit for short common procedures (bilateral myringotomy with tubes and myringoplasty), including tonsillectomy and adenoidectomy. This trend reverses with most standing, regardless of the duration, for removal of nasal foreign body, incision, drainage of neck abscess procedures, and thyroglossal duct cyst excision (Table 1).

**Table 1:** Positioning preferences of 69 otolaryngologists queried OR = Operating room

Procedure	Sitting (%)	Standing (%)	Transport Stretcher (%)	OR Bed (%)
Bilateral myringotomy with tubes	65 (94)	4 (6)	22 (32)	47 (68)
Myringoplasty	66 (96)	3 (4)	18 (26)	51 (74)
Tonsillectomy and adenoidectomy	60 (87)	9 (13)	14 (21)	54 (79)
Direct laryngoscopy and bronchoscopy	69 (100)	0 (0)	7 (10)	62 (90)
Thyroglossal duct cyst excision	2 (3)	67 (97)	3 (4)	66 (96)
Removal of nasal foreign body	4 (6)	65 (94)	21 (31)	47 (69)
Incision and drainage of neck abscess	2 (3)	65 (97)	8 (12)	59 (88)

Nemours internal data was used to calculate average surgical times. Thyroglossal duct cyst excision procedures average 90 minutes. Direct laryngoscopy and bronchoscopy procedures are the next longest, averaging 40 minutes in length. Tonsillectomy and adenoidectomy, and incision and drainage procedures are of intermediate length, averaging 20 to 25 minutes (Table 2). Myringoplasty, bilateral myringotomy with tubes (BMT), and nasal foreign body removal average less than 10 minutes. Preference for the operating room bed was greatest for longer procedures (thyroglossal duct cyst excision). Preference for a stretcher was greatest (31% to 32%) for the two shortest procedures, BMT and removal of nasal foreign body (Table 1). Most surgeons preferred the operating room bed to the transport stretcher, irrespective of procedure length.

**Table 2:** Average institutional surgical times* for common ENT procedures *Nemours internal data obtained from the EMR system. ENT = ear, nose, and throat

Procedure	Average Surgical Time (in minutes)
Bilateral myringotomy with tubes	6
Myringoplasty	5
Tonsillectomy and adenoidectomy	21
Direct laryngoscopy and bronchoscopy	40
Thyroglossal duct cyst excision	90
Removal of nasal foreign body	3
Incision and drainage of neck abscess	22

**Figure 1:**
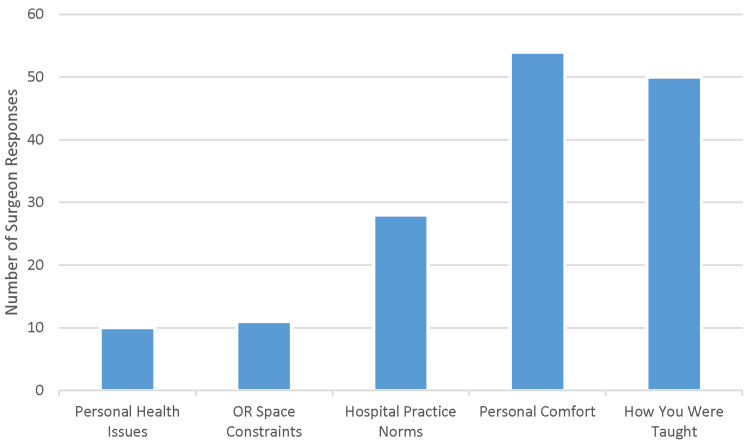
Column chart showing reasons for positioning preferences in the operating room (OR).

Bed preference choices included personal health, operating room space constraints, hospital practice norms, personal comfort, and residency training. Personal comfort (81%) and residency training (75%) were the most frequently selected answers (Figure 1).

Fortunately, pain is infrequent. Four out of five (84%) surgeons denied preexisting neck or back pain, and two out of three (65%) denied developing back pain over the course of their years in practice (Table 3).

**Table 3:** Comparison of preexisting vs. developed pain by body site

Site of Pain	Timing of Pain	
	Preexisting (%)	Developed (%)
Neck	5 (7)	6 (9)
Back	3 (4)	6 (9)
Both	3 (4)	12 (17)
Neither	58 (84)	45 (65)

**Figure 2:**
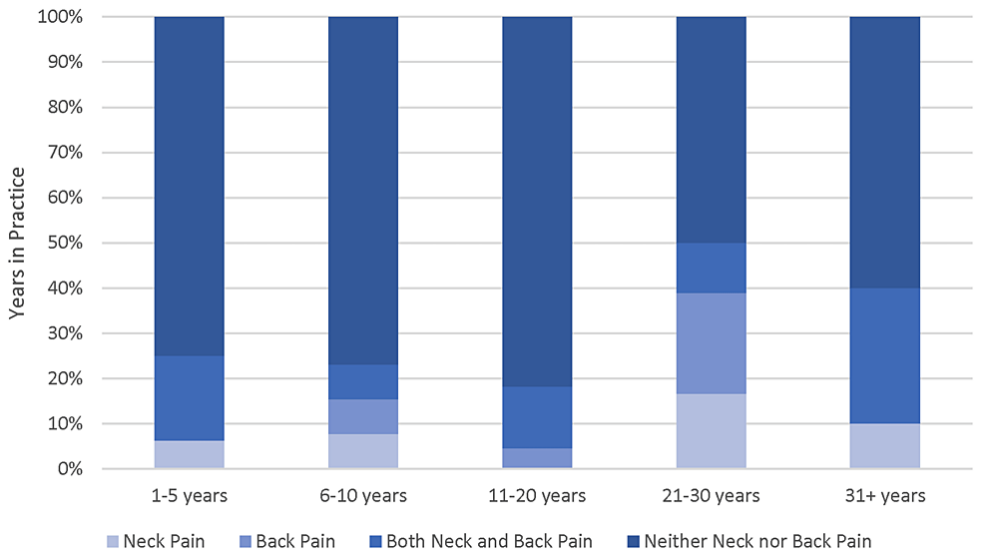
Stacked column chart showing the conditional distribution of pain developed by years in practice.

**Figure 3:**
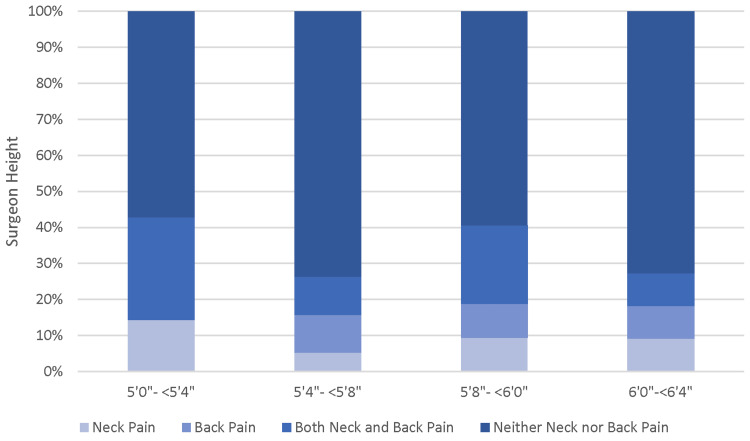
Stacked column chart showing the conditional distribution of pain developed by surgeon height.

Conditional distributions were used to assess the frequency that individuals developed pain during practice for each height category as well as each category of the number of years in practice. Height did not show any correlation. Similarly, there was no linear correlation seen between the report of pain developed and years in practice. However, reports of pain increased for individuals in practice for over 20 years compared with those in practice for under 20 years (Figures 2, 3).

## Discussion

More than 80% of surgeons acknowledge a preference to sit or stand despite operating in two distinctive settings (an outpatient surgical facility vs. a hospital). The goal of an outpatient surgical facility is to reduce cost and maximize efficiency for procedures with predictive resource utilization. Our survey strongly suggests that surgeons’ preferences for their preferred position to operate are developed early during their hospital residency training, and they are reluctant to adapt.

Current research in operating room workflow recognizes that surgeons with repetitive movements in awkward positions frequently develop neck pain, back pain, or both throughout their surgical careers. The more senior surgeons (> 20 years) in our survey confirm a plausible relationship between years in practice and pain; however, causality is not proven. Prior literature has shown underreporting of work-related musculoskeletal injuries throughout all surgical specialties [6].

Pediatric otolaryngology providers demonstrate a persistent preference for sitting for even commonly performed short procedures, e.g., BMT and tonsillectomy, and adenoidectomy. Furthermore, electromyography studies have shown that sitting places increased physical demand on the upper back, neck, and shoulders [7]. This preference may be a legacy from the 20^th^ century when sitting was most common for procedures in otolaryngology (bronchoscopy, esophagoscopy, mastoidectomy, stapedectomy). In a survey study of otolaryngologists in the United Kingdom, Mahalingam et al. show significant focus has been directed toward optimizing patient head position during direct laryngoscopy but failed to generate a practice consensus despite evidence that an optimal position does exist [8]. Furthermore, similar attention has not been directed toward optimizing the surgeon position. Surgeon habits for sitting become ingrained by training and years of practice and are hard to break despite new operating room workflow data. Rimmer et al. show that during endoscopic sinus surgery, standing is associated with increased musculoskeletal pain, and yet 80% of surgeons report standing for those procedures [9]. Access to equipment is also an issue. Aaron et al. show that ergonomically favorable furniture is available to only 15% of surgeons in the office and 28% in the operating room [10].

Recently, the New York Times editorialized that “Sitting is the new smoking!” This opinion is based on data suggesting that standing improves brain activity, which may have a positive impact on cognitive function [3]. When using a standing desk rather than sitting, 37 adults showed increased activity in the alpha band of the parietal region [11]. The Stroop Color and Word Test assess the ability to inhibit cognitive interference in subjects. A smaller Stroop effect was identified in standing participants, who were able to generate the correct answers 0.32 milliseconds faster than sitting participants [12]. Primary school children have shown similar benefits. Traditionally seated students and those using a standing desk for at least an hour per day in a classroom were studied. Working memory and short-term memory tests via the digit span task plus inhibitory control with the Eriksen Flanker Task were assessed. These data show measurable improvements in cognitive function for the children using the standing desk [13].

Our data showing relatively high rates of neck and back pain development for pediatric otolaryngologists is consistent with prior studies. Bolduc‑Bégin et al. show that 88% of surveyed Canadian otolaryngologists reported pain occurring at least sometimes and that half of these reported that their pain limited their daily activities [14]. Similarly, Vijendren et al. show that about half of otolaryngologists surveyed in the United Kingdom experienced work-related musculoskeletal disorders. Most surgeons affected (85%) sought treatment, and one-fifth took time off from work as a result [15]. In the United States, health care is the third most expensive sector for workplace injuries [16]. Babar-Craig et al. demonstrate that up to 53% of otolaryngologists surveyed in the United Kingdom attributed their pain to performing surgeries [17]. Lobo et al. showed a rate of up to 90% for musculoskeletal pain among Spanish otolaryngologists, with a higher prevalence of pain reported among female otolaryngologists compared with their male counterparts [18]. Rodman et al. show that the use of headlamps or loupes conferred an increased risk of musculoskeletal pain in their study [19].

The current survey results indicate that pediatric otolaryngologists have developed their operating room workflow practices based mostly on the factors of prior training and personal comfort. Consensus on sitting versus standing based on procedure type was unsurprising in a small field where many surgeons have been trained by the same surgeon. Ergonomic training remains inconsistent among otolaryngologists. In a 2018 study, Vaisbuch et al. show that only 25% of otolaryngologists reported having undergone ergonomics training [20]. Furthermore, Little et al. previously show that less than 10% of otolaryngologists performing endoscopic sinus surgery described themselves as at least moderately knowledgeable of ergonomic guidelines [21]. However, when otolaryngologists undertook to learn about and apply ergonomic principles, Ho et al. show that 70% observed improvement in their symptoms [22].

Processes tend to develop during early training but may change based on the facility (hospital vs. outpatient surgical facility) and may become very regional when residents stay near their training program. The reasons for surgeon preferences and the potential onset of physical discomfort during decades of surgical practice are rarely discussed. Specifically, do transport and ease of positioning -- “transport stretcher vs. operating room bed” and “sitting vs. standing” -- have any relationship to the length of the procedure and room turnover efficiency?

All surveys are prone to weaknesses. Higher survey response rates are usually equated with greater survey reliability. Nonresponse bias may be a concern in surveys with lower response rates. However, the response rate has been shown to not correlate directly with validity [23]. Holbrook et al. show that surveys with response rates less than 50% and even as low as 5% had only marginally lower accuracy than surveys with much higher response rates [24]. Similarly, Visser et al. demonstrate that surveys with response rates as low as 20% sometimes can be even more accurate than surveys with response rates in the 60% to 70% range [25]. The 38% response rate for this survey is typical of surveys conducted on internal groups. However, survey response may have been blunted by the COVID-19 pandemic that was ongoing at the time. Respondents likely were experiencing a sharp uptick in email volume, and the email survey invitation may have been overlooked. Additionally, other factors like surgeon gender, BMI, and visual acuity that were not included in the survey may also affect positioning preferences, posture, and resultant pain. The use of Loupes or headlights may also affect pain based on the weight of the assistive device and the type used. These are the area for additional study in the future.

## Conclusions

Pediatric otolaryngologists have developed their operating room workflow practices during surgery based mostly on the factors of personal comfort and prior training. However, recent data showing the potential benefits of standing on cognitive function suggest that the current operating room workflow practices for surgeons should be monitored and hopefully can be improved.

## Appendices

Workplace Ergonomics for Pediatric Otolaryngology

Dear Pediatric Otolaryngology Colleagues,

We are asking for 2 minutes of your time to help us better understand surgeon preferences regarding positioning in the operating room. This questionnaire examines the preference for sitting vs. standing and the use of a stretcher vs. OR table for various procedures. We hope to compile and disseminate norms of practice and foster a discussion about optimal OR logistics.

This survey should take no more than 2 minutes to complete. Thank you, in advance, for your participation. If you are interested in the findings, please contact .

The risk to you is minimal. No personal or identifying information, including your email, will be recorded. Participation is voluntary and may be discontinued at any time. For questions about research subjects' rights and concerns, please contact the Nemours Office of Human Subjects Protection at .

Many thanks,

Nicole L. Aaronson, MD & James S. Reilly, MD, Nemours Children’s Health, Wilmington, Delaware

For which pediatric procedures do you sit or stand? (circle)

**Myringotomy with Tubes (BMT): **Sit/Stand

**Myringoplasty: **Sit/Stand

**Tonsillectomy & Adenoidectomy (T&A):** Sit/Stand

**Direct Laryngoscopy/Bronchoscopy: **Sit/Stand

**Thyroglossal Duct Cyst Excision:** Sit/Stand

**Removal of Nasal Foreign Body:** Sit/Stand

**Incision and Drainage (I&D) of Neck Abscess:** Sit/Stand

**For which pediatric procedures do you use the transport gurney vs. the** **operating room bed? (circle)**

**Myringotomy with Tubes (BMT):** Transport Stretcher/OR Bed

**Myringoplasty: **Transport Stretcher/OR Bed

**Tonsillectomy & Adenoidectomy (T&A):** Transport Stretcher/OR Bed

**Direct Laryngoscopy/Bronchoscopy:** Transport Stretcher/OR Bed

**Thyroglossal Duct Cyst Excision:** Transport Stretcher/OR Bed

**Removal of Nasal Foreign Body:** Transport Stretcher/OR Bed

**Incision and Drainage (I&D) of Neck Abscess:** Transport Stretcher/OR Bed

How many pediatric cases (patients < 18) do you perform per month? (circle)

1-20

21-40

41-60

61-80

81+

Where do you perform your pediatric cases? (circle all that apply)

Hospital

Surgicenter

Other

How many years have you been in practice? (circle)

1-5

6-10

11-20

21-30

31+

How tall are you? (circle)

Under 5’0”

5’0” - < 5’4”

5’4” - < 5’8”

5’8” - < 6’0”

6’0” - < 6’4”

6’4” or taller

On what factors are your surgical preferences based? (check all that apply)

Personal health issues

OR space constraints

Hospital practice norms

Personal comfort

How you were taught in the training

Did you have preexisting neck or back pain? (circle)

Neck pain

Back pain

Both neck and back pain

Neither neck nor back pain

Have you developed neck or back pain during your practice? (circle)

Neck pain

Back pain

Both neck and back pain

Neither neck nor back pain

REDCap

projectredcap.org
